# Robust Ensemble Classification Methodology for I123-Ioflupane SPECT Images and Multiple Heterogeneous Biomarkers in the Diagnosis of Parkinson's Disease

**DOI:** 10.3389/fninf.2018.00053

**Published:** 2018-08-14

**Authors:** Diego Castillo-Barnes, Javier Ramírez, Fermín Segovia, Francisco J. Martínez-Murcia, Diego Salas-Gonzalez, Juan M. Górriz

**Affiliations:** Signal Processing and Biomedical Applications (SiPBA), Department of Signal Processing, Networking and Communications, University of Granada, Granada, Spain

**Keywords:** machine learning, ensemble, SVM (support-vector-machine), Parkinson's disease, SPECT (single photon emission computerized tomography), biomarkers, PPMI (Parkinson's progression markers initiative)

## Abstract

In last years, several approaches to develop an effective Computer-Aided-Diagnosis (CAD) system for Parkinson's Disease (PD) have been proposed. Most of these methods have focused almost exclusively on brain images through the use of Machine-Learning algorithms suitable to characterize structural or functional patterns. Those patterns provide enough information about the status and/or the progression at intermediate and advanced stages of Parkinson's Disease. Nevertheless this information could be insufficient at early stages of the pathology. The Parkinson's Progression Markers Initiative (PPMI) database includes neurological images along with multiple biomedical tests. This information opens up the possibility of comparing different biomarker classification results. As data come from heterogeneous sources, it is expected that we could include some of these biomarkers in order to obtain new information about the pathology. Based on that idea, this work presents an Ensemble Classification model with Performance Weighting. This proposal has been tested comparing Healthy Control subjects (HC) vs. patients with PD (considering both PD and SWEDD labeled subjects as the same class). This model combines several Support-Vector-Machine (SVM) with linear kernel classifiers for different biomedical group of tests—including CerebroSpinal Fluid (CSF), RNA, and Serum tests—and pre-processed neuroimages features (Voxels-As-Features and a list of defined Morphological Features) from PPMI database subjects. The proposed methodology makes use of all data sources and selects the most discriminant features (mainly from neuroimages). Using this performance-weighted ensemble classification model, classification results up to 96% were obtained.

## 1. Introduction

Parkinson's Disease (PD) is defined as a chronic, degenerative and neurological disorder that affects the motor system. The origins or triggers that makes appear the PD are still unknown. Several studies have demonstrated this is related to the destruction of pigmented neurons in the substantia nigra (Zetterström et al., 1997; Kordower et al., 2013). Its most frequent symptoms are: tremor, rigidity and bradykinesia, but also cognitive alterations, lack of emotion expressiveness (Pohl et al., 2017) and autonomy problems (Fauci et al., 2008).

One of the most extended tools for PD diagnosis is the use of I123-Ioflupane SPECT (Single Photon Emission Computerized Tomography) images (Neumeyer et al., 1991; Sixel-Döring et al., 2011). These images, also known as FP-CIT or DaTSCAN, make use of the Iodine-123-fluoropropyl-carbomethoxy-3-beta-(4-iodophenyltropane) radio-ligand which presents a high binding affinity for presynaptic dopamine transporters (DAT) in the brain. As a marked reduction in dopaminergic neurons in the striatal region is the most significative feature of PD, DaTSCAN images give us a quantitative measure of the spatial distribution of the transporters in the *striatum*. This information is used in the differentiation of Healthy Control (HC) subjects vs. patients with Parkinson's Disease (PD) (Marek et al., 2001).

However, medical images are not the only effective biomarker that could be used in the diagnosis of PD. In recent years, several works have stated the relation between neurodegenerative disorders and different Biomedical Tests (BT) (Andersen et al., 2017; Dukart et al., 2017; Santiago and Potashkin, 2017). As Handels et al. (2017) points out in its study of Mild Cognitive Impairment (MCI), although some biomarkers could be used for classification purposes (increasing their accuracy in many cases), it is not easy to determine wheter significant improvements are clinically relevant. In fact, we can easily find works with opposing views on the use of biomarkers (Farotti et al., 2017; Mollenhauer et al., 2017) as predictive indicators of PD progression. However, the recent emergence of datasets with biomarkers data and neuroimages has opened up possibilities for the analysis in searching the origins and triggers of the PD progression.

Recently, there has been an increasing interest toward the application of multivariate analysis strategies, such as those based on Machine Learning (ML), to describe between-group differences, in terms of discrimination ability between populations and beyond classical statistical analysis. One of the major problems of ML algorithms is the overfitting problem in high dimensional settings (*d*) with a small sample size (*l*), where the designed classifiers are inevitably over-adjusted to the training set. Unfortunately, in neuroscience this situation is the rule rather than the exception, since the dimensionality of each observation (millions of variables) in relation to the number of available samples (hundreds of acquisitions) implies a high risk of overfitting. This risk can be also explained in terms of the high probability of the training set to be separable by a given surface in high dimensional spaces (Górriz et al., 2017a). The solution to this problem is multi-fold. This situation could be overcome by increasing *l* in resampling methods (i.e., boosting; Hastie et al., 2001) and bagging (Breiman et al., 1984), or by decreasing *d* using feature extraction and selection (FES) approaches (Ramírez et al., 2009; Segovia et al., 2010, 2012; Górriz et al., 2017b). In addition, to preserve complex models from overfitting, some solutions can be adopted that are well-established on cross-validation methods. In this sense, several authors have studied numerous accuracy estimation methods using complex classifiers and cross-validation strategies (Efron, 1983; Kohavi, 1995), i.e., leave-one-out cross-validation.

In neuroimage, multiple Computer-Aided-Diagnosis (CAD) systems have been developed for automatic diagnosis of Parkinson's Disease (Illan et al., 2012; Martinez-Murcia et al., 2014; Augimeri et al., 2016; Segovia et al., 2017b). Most of these systems consist in taking the information collected from medical images: VAF (Voxels-As-Features), textural patterns or morphological features extraction among others. Then, using ML techniques such as Support-Vector-Machines (SVM), Artificial Neural Networks (ANN), Classification trees, Bayesian classifiers, or Kernels; they classify whether a patient is probably suffering the disease, or not, even in its early stages.

Joining these two ideas, we have wondered how to implement an ensemble classification method (Segovia et al., 2014; Badoud et al., 2016) mixing information from clinical tests markers with patterns extracted from images. With this aim, we propose a robust system which combines multiple heterogeneous data sources and weights those that are more discriminative. Mathematically, this work also answers how combinations affects to the final classification and even if multiple sources give us a real significative hint like relationship between heterogeneous sources. We believe that combinations of new promising biomarkers will give us information about indicative factors of Parkinson's Disease progression and diagnosis even when the disease have not clearly manifested yet.

For all individual classifications carried out in this work per feature category (note that none of the classifiers mixes data from heterogeneous information sources), we have made use of linear SVM classifiers (Vapnik, 1998). Additional experiments were also performed using K-Nearest Neighbor (KNN) classifiers (Blanzieri and Melgani, 2008). As the linear SVM showed better results, they were selected as our reference classifiers.

## 2. Materials and methods

### 2.1. PPMI dataset

Data used in the preparation of this article were obtained from the Parkinson's Progression Markers Initiative (PPMI) database (www.ppmi-info.org/data). For up-to-date information on the study, visit www.ppmi-info.org. PPMI—a public-private partnership—is funded by the Michael J. Fox Foundation for Parkinson's Research and funding partners, including all partners listed on www.ppmi-info.org/fundingpartners.

Informed consents to clinical testing and neuroimaging prior to participation of the PPMI cohort were obtained, approved by the institutional review boards (IRB) of all participating institutions. The PPMI obtained written informed consent from all study participants before enrolled in the Initiative. None of the participants were taking any PD medication when they enrolled in the PPMI.

The inclusion criteria adopted in the PPMI cohort study are available in http://www.ppmi-info.org/wp-content/uploads/2014/06/PPMI-Amendment-8-Protocol.pdf. This diagnostical procedure also includes a confirmation step based on imaging but this is not the only test to label a subject. To avoid the possible circularity in results, we have decided not to compare only HC vs. PD patients in our study but HC vs. non-HC subjects instead.

### 2.2. Demographics and descriptive statistics of participants

For this work, we have retrospectively selected the baseline (BL) data available of 388 participants in the PPMI cohort study including Healthy Control subjects (HC), patients with Parkinson's Disease (PD) and those with PD whose scans have no evidence of dopaminergic deficit (SWEDD) (Wyman-Chick et al., 2016). As SWEDD and PD subjects are both considered as patients with Parkinson's Disease, we have included both of them in the same group (PD+SWEDD).

Demographics of all participants have been included in Table 1.

**Table 1:** Demographics.

**Subjects**	**Number**	**Sex [Male—Female]**	**Age [Mean (Std)]**
HC	194	129—65	53.04 (2.27)
PD	168	103—65	53.14 (2.37)
SWEDD	26	17—9	53.21 (2.30)

### 2.3. Image preprocessing

#### 2.3.1. Spatial normalization

All DaTSCAN images have been spatially registered using the SPM (Statistical Parametric Mapping) tool. Specifically, for this work, we have used the SPM12 software package available from: www.fil.ion.ucl.ac.uk/spm/software/spm12/. Its documentation and manuals are also available from this website. Once registration was performed, it was checked that matching between voxels and anatomical structures was unaltered. After being co-registered and averaged, each cerebral image was reoriented into a standard image grid. Obtained images had a dimension of 79 × 95 × 78 voxels and a voxel size of 2.0 × 2.0 × 2.0 mm.

#### 2.3.2. Intensity normalization

Full dataset from the PPMI was used to normalize intensity of each image. An intensity normalization method based on the α-Stable distributions as described in Salas-Gonzalez et al. (2009), Castillo-Barnes et al. (2017) was used for that. This approach has shown itself to be more effective for homogenizing information from SPECT images than other approaches, like the currently widely used intensity normalization based on Binding Ratio or the equivalent Gaussian model, as was demonstrated in Salas-Gonzalez et al. (2013).

Mathematically, intensity normalization based on α-Stable distributions uses a linear transformation as presented in expression (1) with *a* and *b* as follows in (2):

(1) $$\begin{array}{r} Y=aX+b \end{array}$$

(2) $$\begin{array}{r} a=\frac{\gamma^{*}}{\gamma }\;b=\mu^{*}-\frac{\gamma^{*}}{\gamma }\mu \end{array}$$

where γ^*^ and μ^*^ represent the mean of γ (dispersion) and μ (location) parameters, respectively, that are computed for the whole database.

In short, steps to perform intensity normalization using the α-Stable distribution schema can be summarized as follows:

- **Step 1**: A mask is applied to source images in order to consider only voxels in the brain outside the *striatum* (Brahim et al., 2015). This will reduce the computational load without losing too much accuracy.
- **Step 2**: For each image, we compute the histogram of selected voxels in the previous step and fit an α-Stable distribution. We obtain α, β, γ, and δ parameters of each image.
- **Step 3**: Once having all the α-Stable distributions, calculate the γ^*^ and δ^*^ parameters as mean of all γ and δ parameters.
- **Step 4**: Get *a* and *b* values following expression (2).
- **Step 5**: Apply the linear transformation presented in (1).

**Figure 1:**
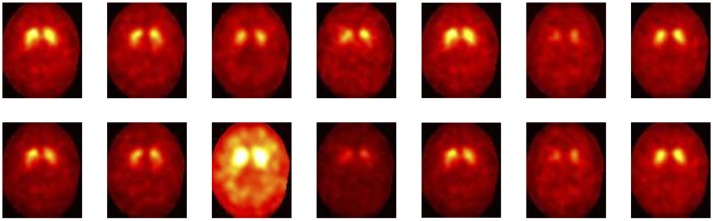
Comparison between intensity normalizated images using the α-Stable normalization procedure **(Up)** and their respective original versions **(Down)**.

A comparison between original and intensity-normalized images is presented in Figure 1.

#### 2.3.3. Region of interest (ROI)

In this work, we considered *striatum* area and non-*striatum* area as significative regions for both intensity normalization and VAF classification purposes.

To get a realistic map from the *striatum*, a segmentation/extraction process was carried out for each image using the AAL (Automated Anatomical Labeling) template (Tzourio-Mazoyer et al., 2002). Thus, we selected regions that compose the *striatum* according to labels from this template.

### 2.4. CSF, plasma, RNA, and serum biomarkers

The PPMI study cohort includes four groups of non-genetic BT: Cerebrospinal-Fluid (**CSF**), **Plasma**, **RNA**, and **Serum** tests. All tests can be downloaded from the PPMI website (www.ppmi-info.org/access-data-specimens/download-data/), specifically from the Biospecimen_Analysis_Results.csv.

Following this idea, one of the objectives of this work is to check if one or more groups of tests can be used, in combination with neuroimaging, to get better classification results. Unfortunately, the PPMI database does not include all tests for each subject. Some tests, specially those referred to Plasma, are not populated enough to avoid small sample size problems. As a simple solution, we have considered only these tests present for a large amount of patients. The list of BT from the Biospecimen_Analysis_Results.csv file and those populated enough are summarized in Table 2.

**Table 2:** List of biomedical tests (BT) included in the PPMI database.

**Group**	**Test name**	**Units**	**Populated**	**Group**	**Test name**	**Units**	**Populated**
CSF	Aβ-42	pg/ml	Yes	RNA	GLT25D1	Ct	Yes
CSF	CSF α-synuclein	pg/ml	Yes	RNA	GUSB	Counts	Yes
CSF	CSF hemoglobin	ng/ml	No	RNA	HNF4A	Ct^(*)^	No
CSF	p-τ181P	pg/ml	Yes	RNA	HSPA8	Ct^(+)^	Yes
CSF	Total-τ	pg/ml	Yes	RNA	LAMB2	Ct^(+)^	Yes
Plasma	Apolipoprotein A1	mg/dL	No	RNA	MON1B	Counts	No
Plasma	EGF ELISA	pg/mL	No	RNA	PGK1	Ct^(+)^	Yes
Plasma	HDL	mg/dL	No	RNA	PSMC4	Ct^(+)^	Yes
Plasma	LDL	mg/dL	No	RNA	PTBP1	Ct^(*)^	No
Plasma	Total cholesterol	mg/dL	No	RNA	RPL13	Counts	Yes
Plasma	Triglycerides	mg/dL	No	RNA	SKP1	Ct	Yes
RNA	ALDH1A1^(+)^	Ct	Yes	RNA	SNCA-007	Counts	Yes
RNA	APP	Ct^(*)^	No	RNA	SNCA-3UTR	Counts	Yes
RNA	C5ORF4	Ct^(*)^	No	RNA	SNCA-E3E4	Counts	Yes
RNA	COPZ1	Ct^(*)^	No	RNA	SNCA-E4E6	Counts	Yes
RNA	DHPR	Counts	Yes	RNA	SOD2	Ct^(*)^	No
RNA	DJ-1	Counts	Yes	RNA	SRCAP	Counts	Yes
RNA	EFTUD2	Ct^(*)^	No	RNA	UBC	Counts	Yes
RNA	FBXO7-001	Counts	Yes	RNA	UBE2K	Ct^(+)^	Yes
RNA	FBXO7-005	Counts	Yes	RNA	WLS	Ct^(*)^	No
RNA	FBXO7-007	Counts	Yes	RNA	ZNF160	Ct^(*)^	No
RNA	FBXO7-008	Counts	Yes	RNA	ZNF746	Counts	Yes
RNA	FBXO7-010	Counts	Yes	Serum	PD2 peptoid	op. density	No
RNA	GAPDH	Ct^(*+)^	Yes	Serum	Serum IGF-1	op. density	Yes

(+) *Tests with two separated repeats*.

(^*^) *Test results in terms of average and standard deviation*.

More specific information about each BT like definitions, its units or extraction procedures are also described at the Biospecimen Analysis Methods section from the https://ida.loni.usc.edu/ website.

### 2.5. Morphological features

Several morphological features were extracted from DaTSCAN images. Then, its performance was compared to a VAF model that uses the *striatum* region as reference. This set of features provides us another classifier for our ensemble model and makes it more robust against missclassifications. Besides, relevant information about structural or functional shapes may be indicative of PD progression (Garg et al., 2015) so it was considered important to include them in this work.

**Figure 2:**
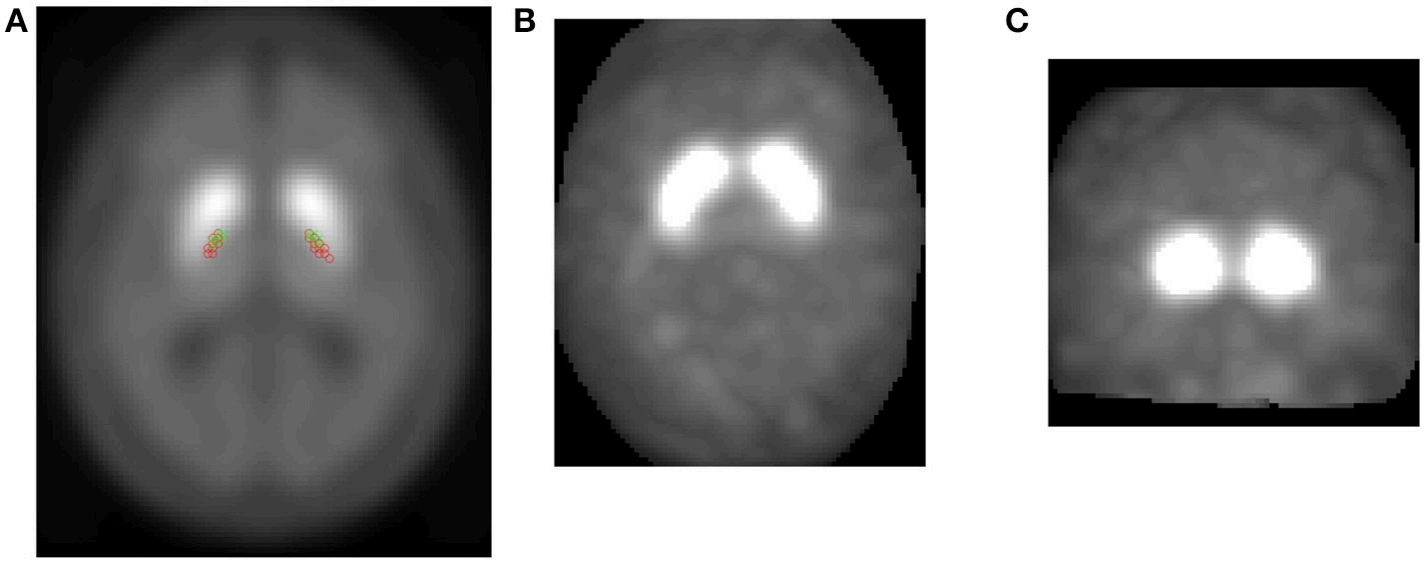
CoM computed for several HC subjects (green) and PD patients (red) in their left/right hemisphere *striatum* region **(A)**. Projections of the *N* most intense voxels obtained from a patient: Axial view **(B)** and coronal view **(C)**.

The morphological features obtained from normalized DaTSCAN images are:

- **Intensity means** - Mean values of intensity in the *striatum* region. It is a 1-by-9 length vector corresponding with: the average intensity of full/left-hemisphere/right-hemisphere voxels in the *striatum* region, the average intensity of the 1% most intense full/left-hemisphere/right-hemisphere voxels in the *striatum* region, the average intensity of the 1% less intense full/left-hemisphere/right-hemisphere voxels in the *striatum* region.
- **Center of mass (CoM)** - Given a particles system, the center of mass of that system is defined as the unique point where the weighted relative position of the distribuited mass sums to zero. In other words, the distribution of particles mass is balanced around the center of mass and the average of the weighted position coordinates of the distribuited mass defines its coordinates. In this work, the same idea has also been used to define a center of intensities instead of mass. To do this, given the relative position (*x, y, z*) of the distributed intensities *I*(*x, y, z*) of all *N*-voxels which forms the *striatum*, we have calculated the exact point where sum of all intensities sums to zero respect that point. *N* has been obtained as the number of voxels that conforms the *striatum* region according to the AAL template. Center of mass has been computed by expression (3) where *I*(*x*_*i*_, *y*_*i*_, *z*_*i*_) represents intensity of the *i*-th voxel with *i* = 1, 2, …, *N* in the (*x*_*i*_, *y*_*i*_, *z*_*i*_) position.
  (3) $$\begin{array}{r} \text{CoM}=\frac{\sum_{i=1}^{N}\left(x_{i},y_{i},z_{i}\right)*I\left(x_{i},y_{i},z_{i}\right)}{\sum_{i=1}^{N}I\left(x_{i},y_{i},z_{i}\right)} \end{array}$$
  Due to *striatum* shape, center of mass has been calculated for each left hemisphere (LH) and right hemisphere (RH) as shown in Figure 2.
- **Projections** - As explained and performed in Segovia et al. (2017a), given a DaTSCAN image, we have projected the *N* most intense voxels in the three directions (*x*, *y*, and *z*). Thus, we obtained three two-dimensional images corresponding to axial projection (calculated as the maximum in the z-axis direction), coronal projection (calculated as the maximum in the y-axis direction) and sagital projection (calculated as the maximum in the x-axis direction). For each image as illustrated in Figure 2, we calculated the following features:
  — **Area** - Number of voxels in the left/right hemisphere projection.
  — **Eccentricity** - Ratio of the distance between the center of the ellipse [with general expression as presented in (4)] and each focus to the length of the semimajor axis *a*.
    (4) $$\begin{array}{r} \frac{{\left(x-x_{0}\right)}^{2}}{a^{2}}+\frac{{\left(y-y_{0}\right)}^{2}}{b^{2}}=1 \end{array}$$
  — **Major axis length** - Length (in voxels) of the major axis (2*a*) of the ellipse that has the same normalized second central moments as the region.
  — **Minor axis length** - Length (in voxels) of the minor axis (2*b*) of the ellipse that has the same normalized second central moments as the region.
  — **Orientation** - Angle between the major axis of the ellipse and the x-axis.
- **Volumes** - A HC subject is expected to present the *striatum* region highly illuminated and approximately homogeneous. For this reason, counting the number of voxels which exceed an intensity threshold may indicate whether a patient meets these specifications. We have calculated the number of voxels which exceeds a certain threshold. This threshold is defined as the 10, 20, 30%,…up to 100% of the averaged intensity value registered at the 1% most intense voxels in the *striatum* region. This measure is expected to be indicative of how quick DATs decrease in the *striatum*.

### 2.6. Ensemble classification

Ensemble classification refers to the process of combining classifiers in order to provide a single and unified classification to an unseen instance (Rokach, 2010). There are two major ways for classifying new instances: fusion and selection. The first approach combines the output of several classifiers whereas selection only selects the output of a single member following a specified and previously defined criterion. In this paper, we have worked with the fusion approach for two reasons: several classifiers were available and none of them affects any individual response of each other.

Assuming that the output of each classifier *i* is a *k*-long vector *p*_*i*, 1_, ⋯, *p*_*i, k*_, where the term *p*_*i, j*_ represents the support that instance **x** belongs to class *j* according to the classifier *i* and it can be assumed (5).

(5) $$\begin{array}{r} \overset{k}{\underset{j=1}{\sum }}p_{i,j}=1 \end{array}$$

In a weighting method, classification results of all members are combined using weights that indicate its effect on the final classification. These weights can be fixed or dynamically determined. A commonly accepted way for this is considering that the weight of each classifier (*w*_*i*_) is proportional to its accuracy performance (α_*i*_) on a validation set (Opitz et al., 1996) as follows in (6):

(6) $$\begin{array}{r} w_{i}=\frac{\left(\alpha_{i}\right)}{\sum_{j=1}^{T}\left(\alpha_{j}\right)} \end{array}$$

Once the weights for each classifier are computed, classes with the highest score are selected by means of expression (7), where *y*_*k*_(**x**) represents the classification of the *k*'th classifier and *g*(*y, c*) is an indicator function defined as (8).

(7) $$\text{Class}(x)=\underset{c_{i}\;\in \;dom(y)}{\text{argmax}}\left(\sum_{k}w_{i}g(y_{k}(x),c_{i})\right)$$

(8) $$g(y,c)=\{\begin{array}{l} 1\;y=c\\ 0\;y\neq c \end{array}$$

Since the weights are normalized and summed up to 1, it is possible to interpret the sum in Equation (7) as the probability that **x**_*i*_ is classified into *c*_*j*_.

When several classifications (but not all) present low accuracies, a sum of several missclassifications can be comparable to good ones. In that case, we need a method that will be able to weight more high scores classifications. To do that, we have used a Windowing technique consisting in increasing the contribution of classifiers with high accuracy rates. This technique is calculated by expression (9), where *f*(α_*i*_) will be a linear, cuadratic or exponential function (among others) as reflected in (10).

(9) $$w(w_{i})=\{\begin{array}{ll} f(\alpha_{i}) & \alpha_{i}\geq 0.5\\ 0 & \alpha_{i}<0.5 \end{array}$$

(10) $$\begin{array}{r} \begin{array}{cc} \text{Linear} & f\left(\alpha_{i}\right)=a\alpha_{i}+b\\ \text{Cuadratic} & f\left(\alpha_{i}\right)=a\alpha_{i}^{2}+b\alpha_{i}+c\\ \text{Exponential} & f\left(\alpha_{i}\right)=ae^{\left(b\alpha_{i}\right)}+c \end{array} \end{array}$$

The only two conditions these expressions should match are: *f*(α_*i*_) = 1 when α_*i*_ = 1 and *f*(α_*i*_) = 0 when α_*i*_ = 0.5, so (10) can be rewritten as (11) assuming that *a* = 1 in the cuadratic and the exponential cases.

(11) $$\begin{array}{r} \begin{array}{cc} \text{Linear} & f\left(\alpha_{i}\right)=2\alpha_{i}-1\\ \text{Cuadratic} & f\left(\alpha_{i}\right)=\alpha_{i}^{2}+0.5\alpha_{i}-0.5\\ \text{Exponential} & f\left(\alpha_{i}\right)=e^{\left(0.9624\alpha_{i}\right)}-1.618 \end{array} \end{array}$$

All individual classifications have been performed using an SVM with linear kernel classifier. Different kernel functions or similarity matrices were not considered necessary as in a multi-modal analisys (Tong et al., 2017; Li et al., 2018). In this case, a simple two-class (binary) classifier is considered as sufficient to separate HC subjects from patients labeled as PD or SWEDD.

### 2.7. Validation

#### 2.7.1. Cross-validation strategy

In order to validate results, dataset has been splited into two groups: a training data group, which we use to train the prediction model, and a test data group, that is then used to measure the classifier's performance through the cross-validation strategy selected. Due to the reduced number of subjects available for each classification, a leave-one-out cross-validation strategy was selected instead of an *N*-fold cross-validation strategy (Kohavi, 1995).

Classification results were analyzed considering the following performance metrics: correct rate or accuracy (Acc), sensitivity or true positive rate (Sens), specificity or true negative rate (Spec) and precision (Prec) as defined in expression (12). *T*_*P*_ is the number of PD patients correctly classified (true positives), *T*_*N*_ is the number of healthy subjects correctly classified (true negatives), *F*_*P*_ is the number of healthy subjects classified as PD (false positives) and *F*_*N*_ is the number of PD patients classified as healthy (false negatives).

(12) $$\begin{array}{r} \begin{array}{ccc} \text{Acc}=\frac{T_{P}+T_{N}}{T_{P}+T_{N}+F_{N}+F_{P}} & \; & \text{Spec}=\frac{T_{N}}{T_{N}+F_{P}}\\ \; & \; & \;\\ \text{Sens}=\frac{T_{P}}{T_{P}+F_{N}} & \; & \text{Prec}=\frac{T_{P}}{T_{P}+F_{P}} \end{array} \end{array}$$

#### 2.7.2. Permutation tests

Non-parametric permutation tests, as referred to in Lehman and Romano (2005), Good (2006), were performed to assess the statistical significance of accuracy rates obtained for each group of patients.

To compute the permutation test, first we have performed a classification with the original labels (diagnoses) of the observations from the PPMI database. This step has resulted in a reference classification result: *R*_*Acc, Original*_. Then, following the process detailed in Ernst (2004), we have randomly rearranged the labels and computed this classification again. The process has been repeated several times until obtaining the distribution of classification results (*R*_*Acc, Per*_*m*__*i*__) for a large number of possible rearrangements (*n* with 1 ≤ *i* ≤ *n*).

Focusing on histogram of all possible results, it would be ideal that the accuracy rates were as far as possible from the center of the distribution. This case means that the original labels give us a better classification result than any other randomized combination of tags and, consequently, our classifier has been able to classify using only representative patterns from the input data. On the contrary, if original labels had given us a result near the central point of the histogram (in which is suppposed to have got most of the cases), it would be a sign that our classifier has not been able to find a significative pattern. In this last case, missclassification mistakes would be significant.

### 2.8. General diagram

**Figure 3:**
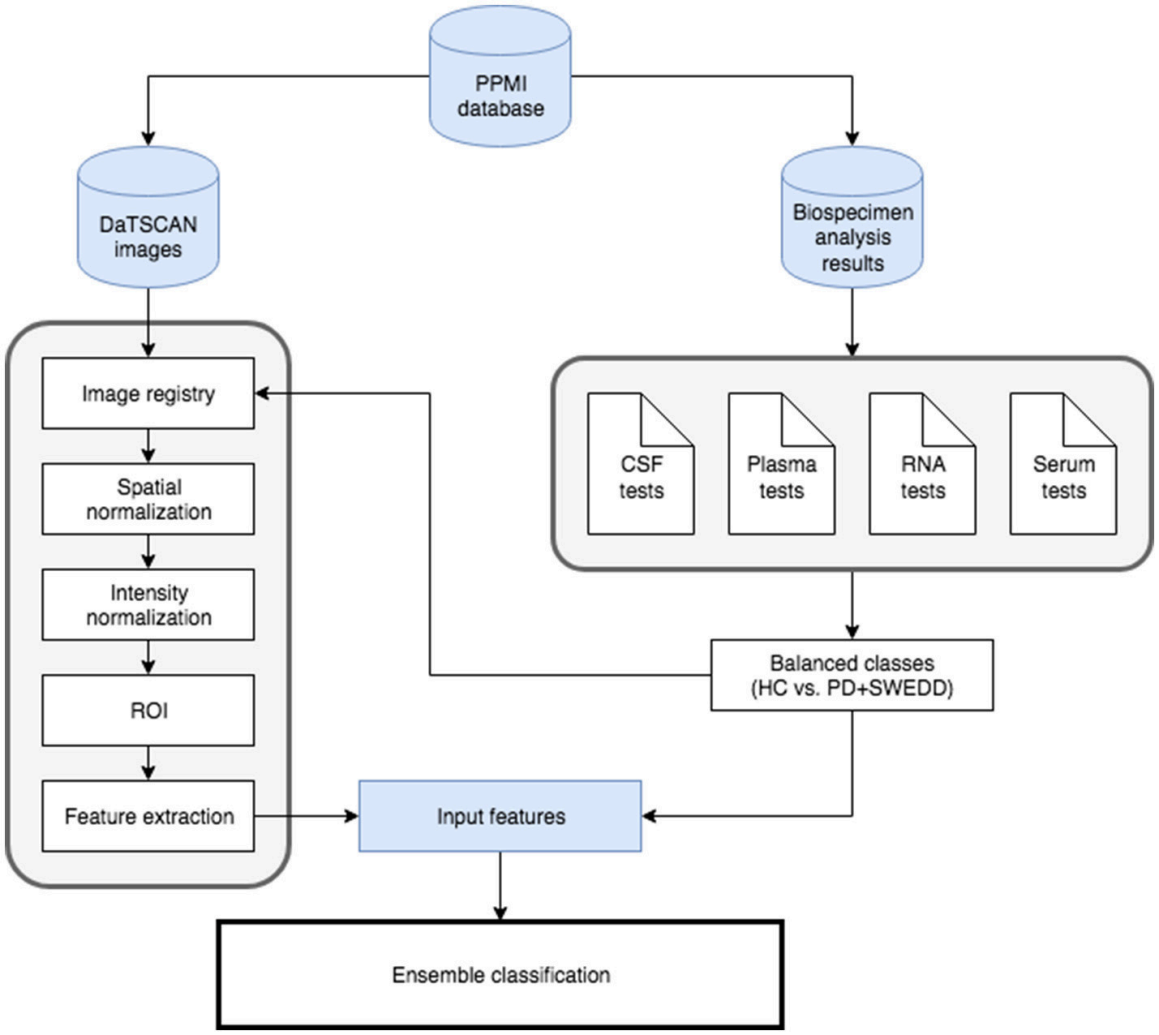
General diagram of work.

**Figure 4:**
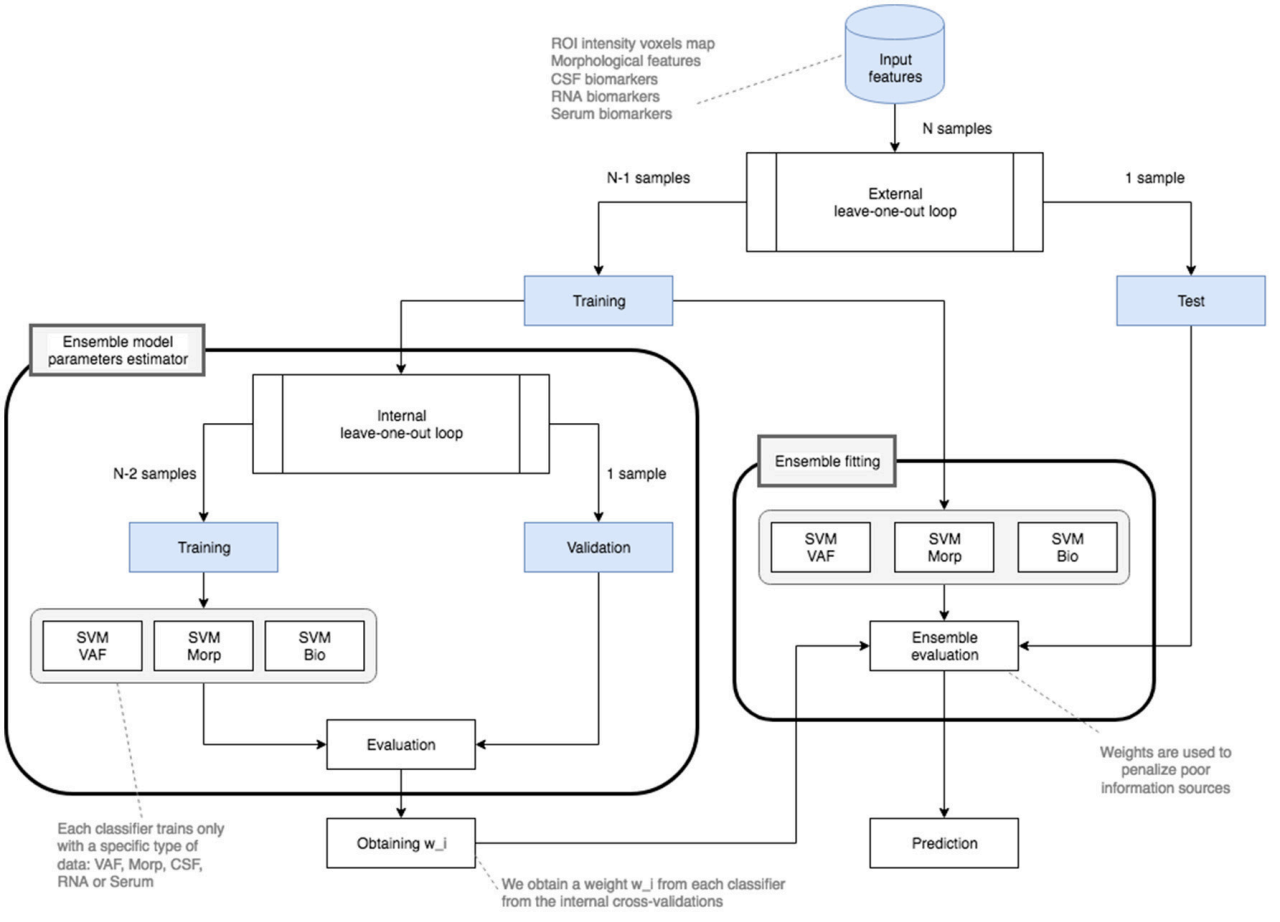
Ensemble classification flowchart for an experiment with *N* balanced subjects.

Diagram including all steps has been depicted in Figure 3. Detailed flowchart showing the ensemble classification model has also been included in Figure 4. This flowchart is similar to the presented in Dai et al. (2012) and consists in the use of two classification loops:

- First of all, preprocessed input features are splitted into two parts: a training data set and a test data set.
- As we are using a leave-one-out cross-validation schema for both external and internal loops, the first training data set consists of *N*−1 samples whereas the test set only presents 1 sample.
- The training set is used for two loops:
  - A nested loop which gets the accuracies of several linear SVM classifiers. It uses *N*−2 samples to obtain a data model and makes a cross-validation with the remaining sample. This will result into a *w*_*i*_ weight obtained evaluating each individual (**VAF**, **Morp**, and biomedical tests -**CSF**, **Plasma**, **RNA**, and **Serum**-) classifier.
  - An external loop that fits a model for each data source. This schema uses the original training data with *N*−1 samples for fitting the model as reflected in Figure 4.
- Once all the models are created and evaluated on the Test data, and when the nested loop returns the weights *w*_*i*_, the ensemble classification is performed. For that, the main loop, which also follows a leave-one-out validation schema, applies the windowing technique proposed and obtains the fusion parameters (accuracy, sensitivity, specificity, and precision) using the remaining test sample.

Note that different kind of classifiers and cross-validation schemas may be used instead of linear SVM classifiers and/or leave-one-out due to the flexibility of our proposal.

## 3. Results

The proposed methodology has been tested using 388 different SPECT images (194 HC, 168 PD, and 26 SWEDD subjects) in baseline (BL) as cited in Table 1.

**Figure 5:**
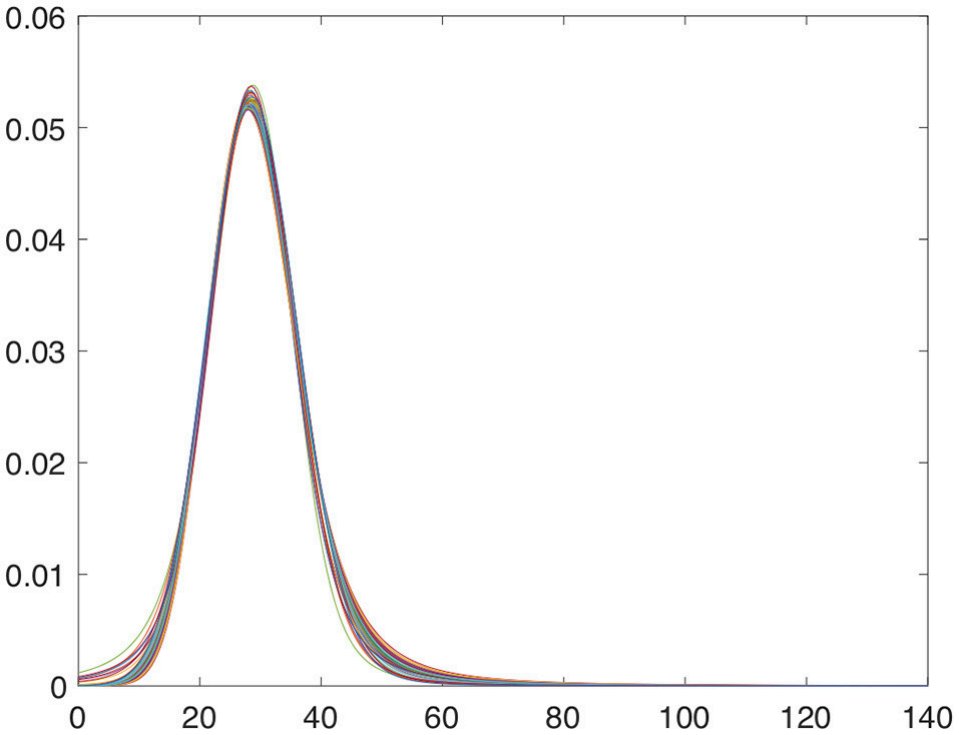
Intensity distribution of the first 50 DaTSCAN images after applying the α-Stable intensity normalization.

All images have been spatially normalizated and the intensity normalization approach explained in section 2.3.2 has also been applied. After intensity normalization, histograms of the intensity values present an α-Stable distribution centered on location δ = 28.42 and with dispersion γ = 5.41. Representation of final intensity distributions are shown in the Figure 5.

*Striatum* volume for VAF was calculated using the AAL template including both *caudate nucleus* and *putamen* areas. Nevertheless, for reasons of anatomical relationship with the nigrostriatal pathway, the following structures were also included: *globus pallidus, thalamus, olfactory cortex, amygdala, hippocampus, inferior temporal gyrus*. Consequently, the final volume considered as ROI contained *N* = 21, 981 voxels in total.

A total of 68 BT were processed from the Biospecimen_Analysis_Results.csv file. Some of these tests are given in terms of average and standard desviation as reflected in Table 2. Despite of this, all values were included as input features in a matrix utilized for classification. Note that, due to the lack of some medical tests findings for some patients, we have decided to restrict the number of BT under study from 68 to 39.

To further reduce the number of experiments not providing relevant information to the ensemble methodology, a rank of features procedure based on the use of Welch's *U*-Test was performed for the biomedical tests. Thus, we have estimated the significance of each biomarker according to its most significative value (a minor *p*-value). As we can check in Table 3, **Plasma** tests do not contribute to the possible separation between classes in comparisson with the other biomedical tests. This result, in addition to the small number of **Plasma** features available, reinforces the idea of discarding all combinations containing **Plasma** tests from posterior analyses. Final experiments combining all representative groups of BT, balanced and large enough, are presented in Table 4 where it was also indicated the number of features considered for each data source. Even in this point, the list of final biomarker features could have been more reduced by ranking the features and selecting those ones with a better performance. However, as there were not much clinical information available for all the patients, we finally decided using as many tests as possible and the feature selection were performed only regarding to their number.

**Table 3:** Welch's *U*-Test analysis for CSF, Plasma, RNA, and Serum.

**Type**	**Welch's *U*-Test most significative *p*-value**	**Cases with (*p*−*value* < 0.05)**
CSF	0.0017	CSF α-synuclein, p-τ181P, Total-τ
Plasma	0.4887	-
RNA	0.0052	ALDH1A1, GAPDH, PGK1
Serum	0.0756	-

**Table 4:** List of experiments with all representative groups of cases, balanced, and large enough.

**Experiment**	**No. subjects**	**VAF 21,981 voxels**	**Morp 55 values**	**CSF 4 BT results**	**RNA 34 BT results**	**Serum 1 BT result**
1	334	✓	✓	✓		
2	150	✓	✓	✓	✓	
3	306	✓	✓	✓		✓
4	148	✓	✓	✓	✓	✓
5	150	✓	✓		✓	
6	148	✓	✓		✓	✓
7	310	✓	✓			✓

As represented in Figure 3, once data sources have been properly pre-processed, the next step is to classify/diagnose subjects through the ensemble classification model proposed. For that, the nested loop in Figure 4 consists of SVM with linear kernel classifiers for **VAF**, **Morp**, **CSF**, **RNA**, **Serum**. Then, in order to validate results of each classifier, a leave-one-out validation strategy has been carried out. Individual accuracy, sensitivity, specificity and precision are summarized in Table 5. Note that for **VAF**, only voxels from *Striatum* area were considered as input features.

**Table 5:** Classification results (individual classifications using linear SVM classifiers).

**Experiment**	**Parameter**	**VAF (%)**	**Morp (%)**	**CSF (%)**	**RNA (%)**	**Serum (%)**
1	Accuracy	82.93	88.32	52.99	-	-
	Sensitivity	84.43	87.43	73.05	-	-
	Specificity	81.44	89.22	32.93	-	-
	Precision	81.98	89.02	52.14	-	-
2	Accuracy	96.00	90.67	56.67	58.67	-
	Sensitivity	96.00	90.67	74.67	58.67	-
	Specificity	96.00	90.67	38.67	58.67	-
	Precision	96.00	90.67	54.90	58.67	-
3	Accuracy	96.73	91.50	53.27	-	51.96
	Sensitivity	96.08	91.50	70.59	-	24.18
	Specificity	97.39	91.50	35.95	-	79.74
	Precision	97.35	91.50	52.43	-	54.41
4	Accuracy	96.62	89.86	55.41	48.65	52.03
	Sensitivity	95.95	89.19	74.32	47.30	24.32
	Specificity	97.30	90.54	36.49	50.00	79.73
	Precision	97.26	90.41	53.92	48.61	54.55
5	Accuracy	96.00	91.33	-	48.00	-
	Sensitivity	96.00	92.00	-	49.33	-
	Specificity	96.00	90.67	-	46.67	-
	Precision	96.00	90.79	-	48.05	-
6	Accuracy	95.95	90.54	-	52.03	52.70
	Sensitivity	94.59	89.19	-	50.00	18.92
	Specificity	97.30	91.89	-	54.05	86.49
	Precision	97.22	91.67	-	52.11	58.33
7	Accuracy	96.45	92.26	-	-	52.58
	Sensitivity	95.48	92.90	-	-	23.87
	Specificity	97.42	91.61	-	-	81.29
	Precision	97.37	91.72	-	-	56.06

**Figure 6:**
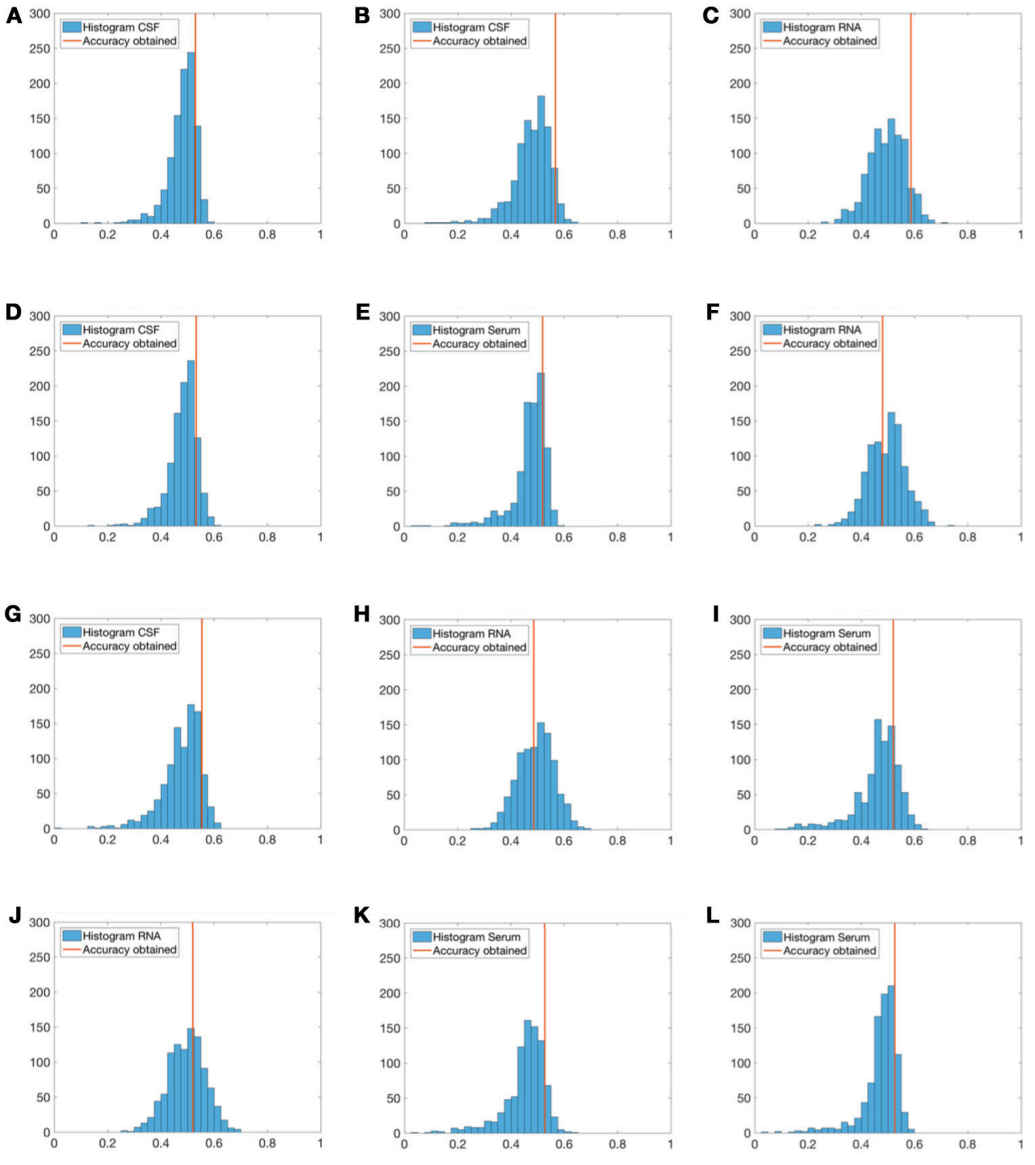
Histograms of the accuracy rates achieved by using randomly generated label sets (1, 000 repetitions) and the proposed method. Red lines represent the accuracy obtained for each group of tests. Experiment 1—CSF **(A)**, Experiment 2—CSF **(B)**, Experiment 2—Serum **(C)**, Experiment 3—CSF **(D)**, Experiment 3—RNA **(E)**, Experiment 5—RNA **(F)**, Experiment 4—CSF **(G)**, Experiment 4—RNA **(H)**, Experiment 4—Serum **(I)**, Experiment 6—RNA **(J)**, Experiment 6—Serum **(K)**, and Experiment 7—Serum **(L)**.

For greater reliability, a non-parametric permutation test was performed for all sets of medical biomarkers (**CSF**, **RNA**, and **Serum**) to assess the statistical difference between accuracy rates obtained using the SVM with linear kernel classifiers. 1, 000 sets of random diagnostic labels (each of them with the same lenght as the original) were generated, then each classifier was trained with these random labels and the accuracy estimated. Histograms of *p*-value results were generated, and subsequently, compared to SVM original results as shown in Figure 6.

A one-sample *t*-test was also performed a posteriori. As shown in Table 6, results rejected the null hypotheses. This means, the data in each permutation test does not come from a normal distribution with mean equal to the accuracy obtained by its respective original classification.

**Table 6:** One-sample *t*-Test performed to discard the null hypotheses.

**Experiment**	**Type**	**Null hypotheses**	***p*-value**	**Confidence interval**	**Stats**
1	CSF	Rejected	≈0	[0.4782, 0.4847]	tstat = −29.2183 sd = 0.0525
2	CSF	Rejected	≈0	[0.4734, 0.4821]	tstat = −40.2423 sd = 0.0699
	RNA	Rejected	≈0	[0.4916, 0.5001]	tstat = −42.1418 sd = 0.0681
3	CSF	Rejected	≈0	[0.4789, 0.4855]	tstat = −29.6297 sd = 0.0539
	Serum	Rejected	≈0	[0.4236, 0.4416]	tstat = −18.9669 sd = 0.1451
4	CSF	Rejected	≈0	[0.4724, 0.4816]	tstat = −32.7918 sd = 0.0743
	RNA	Rejected	≈0	[0.4894, 0.4979]	tstat = 3.3227 sd = 0.0682
	Serum	Rejected	≈0	[0.3925, 0.4141]	tstat = −21.2206 sd = 0.1743
5	RNA	Rejected	≈0	[0.4899, 0.4983]	tstat = 6.5119 sd = 0.0685
6	RNA	Rejected	≈0	[0.4918, 0.5005]	tstat = −10.8945 sd = 0.0699
	Serum	Rejected	≈0	[0.3841, 0.4048]	tstat = −25.1350 sd = 0.1668
7	Serum	Rejected	≈0	[0.4255, 0.4435]	tstat = −19.9518 sd = 0.1447

Once nested loop is fully iterated, individual classications are performed and the ensemble classification methodology can be carried out.

**Figure 7:**
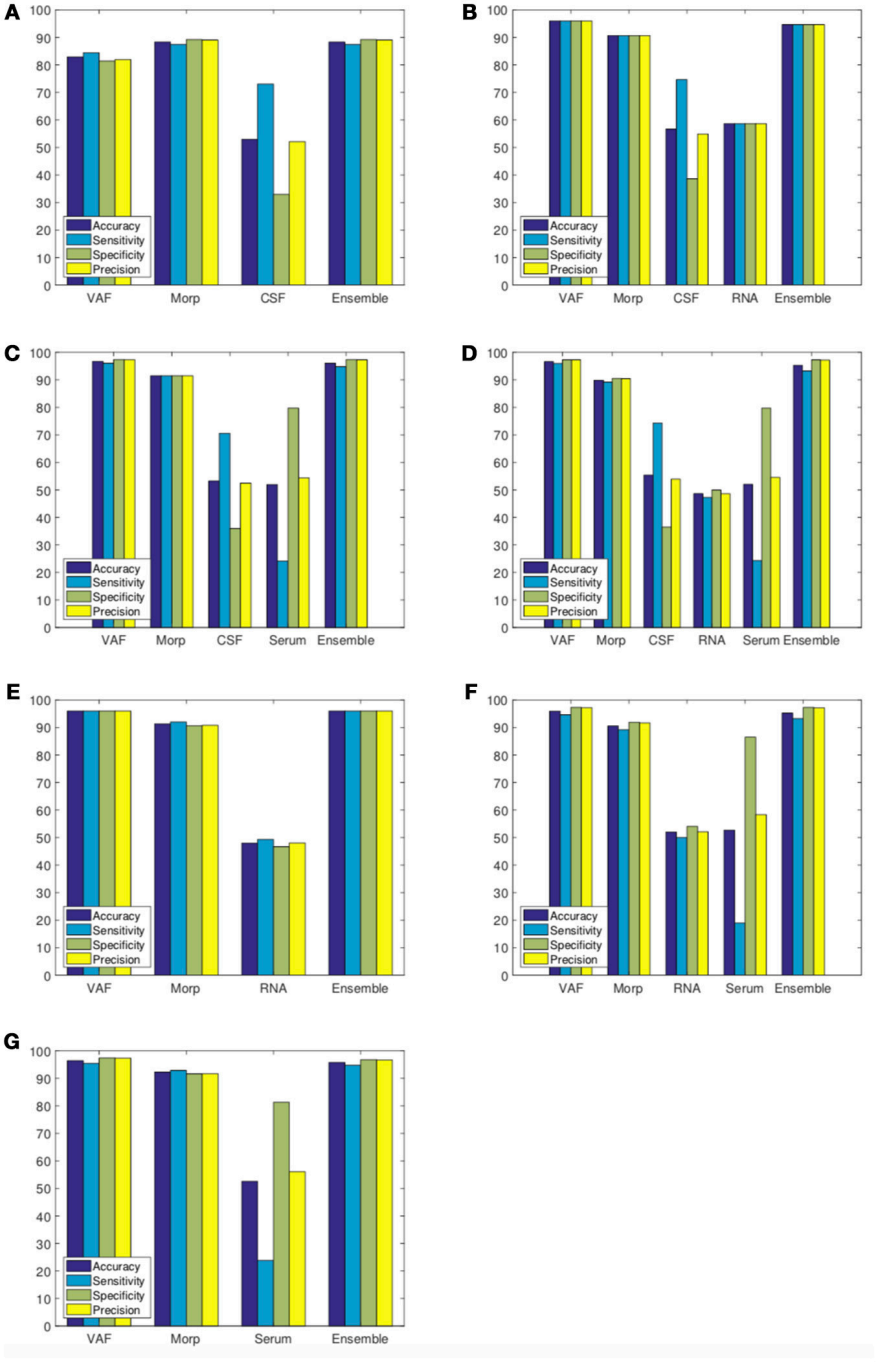
Classification results considering Ensemble Classification. Experiment 1 **(A)**, Experiment 2 **(B)**, Experiment 3 **(C)**, Experiment 4 **(D)**, Experiment 5 **(E)**, Experiment 6 **(F)**, and Experiment 7 **(G)**.

Different ensemble classification approaches, most of them based on Performance Weighting (PW), have been performed as shown in Table 7. Final results including individual classifications and the ensemble fusion method are presented in Figure 7.

**Table 7:** Classification results—Accuracy obtained from different ensemble methods.

**Experiment**	**Majority voting (MV) (%)**	**Performance weighting (PW) (%)**	**PW with linear windowing (*ax*+*b*) (%)**	**PW with cuadratic windowing (*ax*^2^+*bx*+*c*) (%)**	**PW with exponential windowing (*ae*^*bx*^+*c*)**	**Hyperplane distance (%)**
1	85.63	85.63	88.02	88.32	85.63	86.83
2	88.67	94.67	94.67	94.67	94.67	91.33
3	85.62	95.75	95.42	96.08	95.75	93.79
4	83.11	93.92	94.59	95.27	89.86	88.51
5	93.33	93.33	96.00	96.00	94.00	92.67
6	77.70	93.24	94.59	95.27	95.27	90.54
7	85.68	92.76	93.88	94.27	92.53	90.61

Although all classifications were performed using linear SVM classifiers, as mentioned in the 1, a second battery of simulations was also performed making use of K-Nearest Neighbor (KNN) classifiers. Results of these simulations have been included as Supplementary Material. Due to the worse classification rates obtained with this kind of classifiers, their use was discarded.

**Figure 8:**
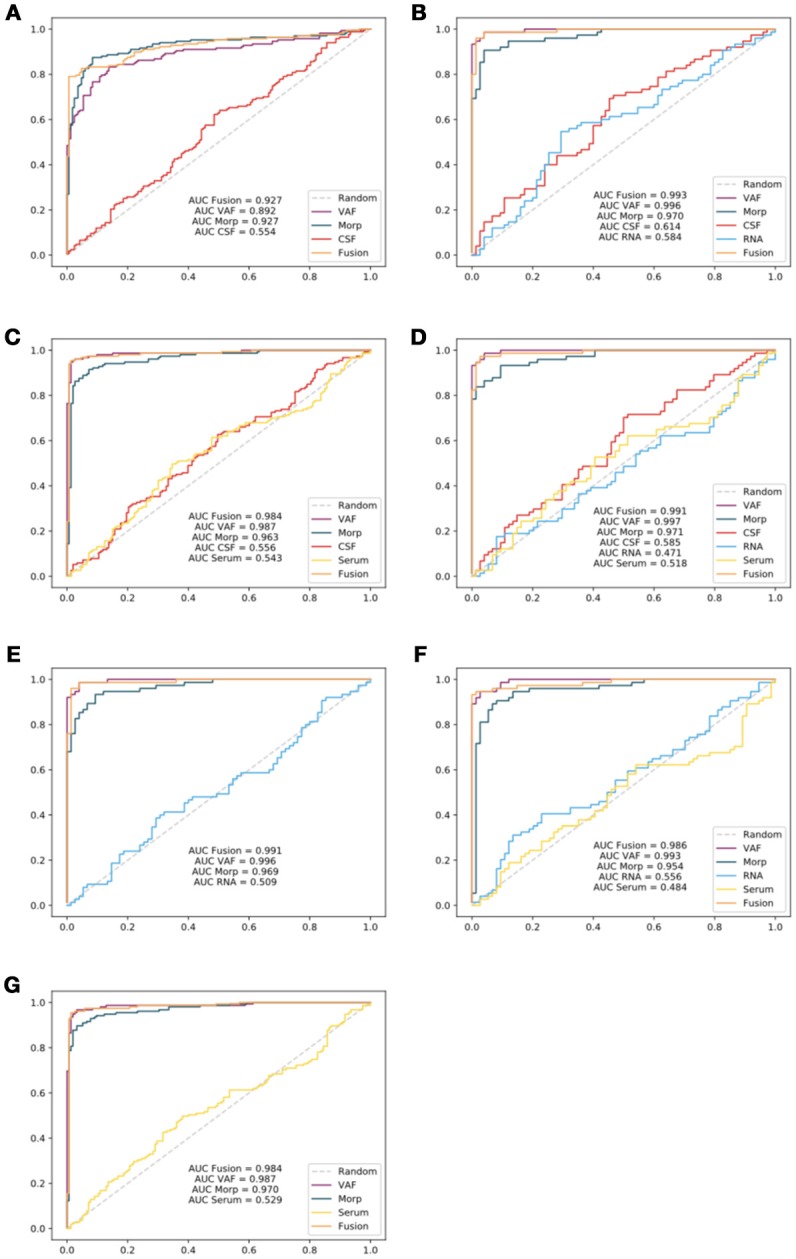
ROC curves generated for each experiment: Experiment 1 **(A)**, Experiment 2 **(B)**, Experiment 3 **(C)**, Experiment 4 **(D)**, Experiment 5 **(E)**, Experiment 6 **(F)**, and Experiment 7 **(G)**. Area Under the Curve (AUC) parameters for all experiments have also been reflected in the curves.

Finally, to highlight the difference between sets of medical tests (**CSF**, **RNA**, and **Serum**), image features and the ensemble model that combines all of them; a further comparison was performed by means of the Receiver Operating Characteristic (ROC) curves (Zweig and Campbell, 1993) for the seven experiments (see Figure 8).

## 4. Discussion and conclusions

Despite the interest, many questions remain open surrounding the topic of Parkinson's Disease. As a general view (Meireles and Massano, 2012), it is expected that combination of different data sources will give us the necessary keys to determine precisely which are the origins and predictive factors of PD.

Although medical science has begun to consider neuroimaging analysis as the reference test in the diagnosis of Parkinson's Disease (Salvatore et al., 2014), results like VAF analysis with an accuracy up to 95% in many studies are hardly able to be improved even by employing advanced techniques of Machine Learning. In these terms, this work presents many significative strenghts: a robust classification methodology that combines an effective intensity normalization technique based on the use of α-Stable distributions; a classification schema which maximizes models obtained for each group of markers; a multimodal CAD system that combines multiple heterogeneous data sources and an ensemble classifier that selects the most reliable characteristics from input sources as indicated in Tables 5, 7.

If we compare our final proposal (*Performance Weighting with Cuadratic Windowing*) with the baseline method (*Majority Voting*) as shown in Table 7, we obtain an averaged improvement of 7.46%. This fact reinforces our main idea: if we use better (more discriminative) biomarkers, ensemble classification rates will increase. As it can be checked, biomedical tests with poor classification rates in the internal cross-validation loop are strongly penalized by the windowing technique so the final classification (external loop) makes a poor use of them. In fact, for this work, only image-based classifiers (**VAF** and **Morp**), with averaged accuracies of 94.38 and 90.64%, respectively, have proven to be good enough to the final ensemble classification. Such importance is explained through the cuadratic windowing method described in (11). For example, if we compare results from experiment 2, **CSF** and **RNA** tests resulted in a weight of *w*_*CSF*_ = 0.10 and *w*_*RNA*_ = 0.14, whereas **VAF** obtained a weight of *w*_*VAF*_ = 0.90 and **Morp** was *w*_*Morp*_ = 0.77. As markers based on image presented higher weights^1^, it results in a final classification result similar to them.

For this study, results issued by the Welch's *U*-Test are consistent with the current state-of-the-art as reflected in Gallegos et al. (2015), Klettner et al. (2016), Xu et al. (2017), Hu et al. (2017), Vanle et al. (2017), and Abbasi et al. (2018), particulary for **CSF** and **RNA** tests (**CSF Alpha-synuclein**, **p-**τ**181P**, **Total-**τ, and **GAPDH**). We confirm this hypothesis as we obtain better ensemble classification results when those biomarkers are included in our multimodal experiments. However, as the weights obtained from these biomedical tests were rather small, the ensemble methodology has not been able to take advantage of them. Only features with individual classification rates equal to or above 50% are useful for our classification purposes. Though it could be seen as a disadvantage, discarding group of tests whose are not well-related to the disease prognosis also decreases computation costs and let us to center our focus on those biomedical tests that really matter.

Experiments involving **Serum** tests presented high accuracy rates. Nevertheless, they do not provide a reliable source of information as reflected in ROC curves (Figure 8) with AUC values for ensemble model substantially below single **VAF** or **Morp**. A direct consequence of this fact may be the need to discard this type of tests defined by the PPMI in a previous phase for future works.

In view of the obtained results, and as we can see in Figure 6 in relation to biomedical tests, no general conclusions can be drawn for experiments that have presented *p*-values above 5% significance level (none of the experiments presented a *p*-value under 0.05 and only experiment 2, and experiment 4 with *p*-values between 0.05 and 0.1). In comparison with Welch's *U*-Test in Table 3, **RNA** and **CSF** features with *p*-values below 0.05 should be enough to discern between PD and HC subjects. However, this idea is not reflected in the permutation tests. The main reason could be the small sample size of groups: if distribution variance of accuracies increases, *p*-value is also increased.

This CAD system can be used to determine an early diagnosis or evolution of Parkinson's Disease. Subjects information for the last 5, 10, 15, or 20 years may be used to determine how disease has progressed. In this sense, if we could work using longitudinal information, we will face up to Parkinson's Disease from a different perspective: not only confirming if a subject shows signs of suffering the neurological disorder but also if that person may develop this pathology in the future.

Though there are not many works related to the use of ensemble classification methodologies for the study of neurodegenerative diseases, the use of Neural Networks or Tree-Based Models with different kind of classifiers as ensemble approaches are quite prominent. Works like presented in Khan et al. (2016) and Li and Wang (2017) which made use of datasets based on speech recordings were able to reach accuracies up to 90%. Other works like (Challa et al., 2016) also combine different imaging biomarkers with biomedical tests to make a model of the disease. In this sense, we could also cite the work presented in Latourelle et al. (2017) which performs a longitudinal study of Parkinsonism based on the use of different clinical, molecular and genetic data. The small size of the dataset used in some of these studies and the computation costs in several cases may be some of the strongest disadvantages with respect to our proposal. Only the proposal presented in Ramírez et al. (2018), for Alzheimer's Disease diagnosis, makes use of a multi-level robust ensemble classification model.

One last point to close this section 4 has a close relation to the most important problem we have had to face up: the lack of all medical tests results for all patients. Although our study was designed to work with the entire PPMI database, due to the lack of all medical tests our experiments have not been able to count on all subjects. In this sense, three main ideas have been suggested for future works:

- The inclusion of Missing Data (MD) techniques which are already being implemented in fields like wireless networks or data mining (Magán-Carrión et al., 2015).
- Add new promising biomarkers as referred on Saiki et al. (2017) and Delgado-Alvarado et al. (2017) or study relations between existing ones (Constantinides et al., 2017; Fereshtehnejad et al., 2017).
- Include new image markers as stated in Saeed et al. (2017) or make use of different image sources combined as done by Segovia et al. (2017b).
- The design of a dynamic feature selection procedure for the internal loop which may be also used by the external ensemble loop.

In regarding to its easy adaptation, the proposed methodology presented in this work can also be used for many other databases such as ADNI (http://adni.loni.usc.edu/) or DIAN (https://dian.wustl.edu/). Moreover, the extension of this proposal with the inclusion of procedures for semi-supervised learning or the use of data imputation techniques will face up with the lack of complete tests.

## Author contributions

DC-B, JR, and DS-G: conception or design of the work. DC-B, FS, and FM-M: data collection. DC-B, JR, and DS-G: data analysis and interpretation. DC-B, JR, JG, and DS-G: drafting of the article. JR, FS, FM-M, DS-G, and JG: critical revision of the article. DC-B, JR, DS-G, and JG: major revision of the article.

### Conflict of interest statement

The authors declare that the research was conducted in the absence of any commercial or financial relationships that could be construed as a potential conflict of interest.

## Footnotes

^1^A further summary of weights obtained in our experiments have been included as Supplementary Material.
