# Supplementary material for: Robust Ensemble Classification Methodology for I123-Ioflupane SPECT Images and Multiple Heterogeneous Biomarkers in the Diagnosis of Parkinson's Disease
Source: Front Neuroinform. 2018 Aug 14;12:53. doi: 10.3389/fninf.2018.00053 (PMC6102321; doi:10.3389/fninf.2018.00053)
Supplement: Supplementary file 1 [file Data_Sheet_1.PDF]

Supplementary Material:
Robust ensemble classification methodology for I123-Ioflupane SPECT images and multiple heterogeneous biomarkers in the diagnosis of Parkinson's Disease

1 SUPPLEMENTARY DATA

For this work we have made use of linear SVM classifiers for individual classifications. However, as a way to compare our results with another approaches, we have also perfomed all experiments using K-Nearest Neighbor (KNN) classifiers as mentioned in the **Introduction** section.

After previous tests trying different k values for KNN classifiers, we have decided to carry out all experiments in this section using $k = 3$. Individual classifications and accuracy results obtained from different ensemble methods using KNN classifiers have been summarized in Tables S1 and S2 respectively.

Finally, although computation of the weights have been detailed in the **Results** section, a brief of the weights obtained using the PW with cuadratic windowing approach for all experiments have been included. Weights results are shown in Tables S3 when using linear SVM, and S4 when using KNN.

2 SUPPLEMENTARY TABLES AND FIGURES

2.1 Tables

Experiment	Parameter	VAF	Morp	CSF	RNA	Serum
1	Accuracy	79.94%	84.43%	51.80%	-	-
	Sensitivity	83.83%	80.84%	51.50%	-	-
	Specificity	76.05%	88.02%	52.10%	-	-
	Precision	77.78%	87.10%	51.81%	-	-
2	Accuracy	88.00%	84.67%	40.00%	54.67%	-
	Sensitivity	92.00%	86.67%	42.67%	53.33%	-
	Specificity	84.00%	82.67%	37.33%	56.00%	-
	Precision	85.19%	83.33%	40.51%	54.79%	-
3	Accuracy	86.93%	87.58%	54.58%	-	49.35%
	Sensitivity	90.85%	85.62%	54.90%	-	48.37%
	Specificity	83.01%	89.54%	54.25%	-	50.33%
	Precision	84.24%	89.12%	54.55%	-	49.33%
4	Accuracy	89.86%	86.49%	39.86%	57.43%	47.97%
	Sensitivity	95.95%	82.43%	40.54%	52.70%	48.65%
	Specificity	83.78%	90.54%	39.19%	62.16%	47.30%
	Precision	85.54%	89.71%	40.00%	58.21%	48.00%
5	Accuracy	88.67%	85.33%	-	52.00%	-
	Sensitivity	92.00%	85.33%	-	56.00%	-
	Specificity	85.33%	85.33%	-	48.00%	-
	Precision	86.25%	85.33%	-	51.85%	-
6	Accuracy	89.86%	86.49%	-	50.68%	50.68%
	Sensitivity	94.59%	86.49%	-	48.65%	51.35%
	Specificity	85.14%	86.49%	-	52.70%	50.00%
	Precision	86.42%	86.49%	-	50.70%	50.67%
7	Accuracy	86.77%	87.42%	-	-	48.71%
	Sensitivity	91.61%	85.81%	-	-	47.10%
	Specificity	81.94%	89.03%	-	-	50.32%
	Precision	83.53%	88.67%	-	-	48.67%

Table S1. Classification results (individual classifications using linear KNN classifiers).

Experiment	Majority voting (MV)	Performance weighting (PW)	PW with linear windowing $(ax+b)$	PW with cuadratic windowing (ax^2+bx+c)	PW with exponential windowing $(ae^{bx}+c)$	Hyperplane distance
1	82.93%	82.93%	84.43%	**84.43%**	82.93%	50.00%
2	74.67%	84.67%	83.33%	82.67%	84.67%	50.00%
3	78.10%	89.54%	89.54%	**89.54%**	89.54%	50.00%
4	75.68%	85.14%	87.84%	86.49%	87.84%	50.00%
5	84.67%	84.67%	86.67%	**88.00%**	84.67%	50.00%
6	81.76%	89.19%	89.19%	**89.86%**	87.16%	50.00%
7	85.48%	85.48%	86.77%	**86.77%**	86.77%	50.00%

Table S2. Classification results - Accuracy obtained from different ensemble methods. (simulation results using KNN classifiers).

**Experiment**	W_{VAF}	W_{Morp}	W_{CSF}	W_{RNA}	W_{Serum}
**1**	0.60	0.72	0.05	-	-
**2**	0.90	0.77	0.10	0.14	-
**3**	0.92	0.79	0.05	-	0.03
**4**	0.92	0.76	0.08	0.00	0.03
**5**	0.90	0.79	-	0.00	-
**6**	0.90	0.77	-	0.03	0.04
**7**	0.91	0.81	-	-	0.04

Table S3. Weights obtained from each data source when using PW with cuadratic windowing and linear SVM classifiers.

**Experiment**	W_{VAF}	W_{Morp}	W_{CSF}	W_{RNA}	W_{Serum}
**1**	0.54	0.63	0.03	-	-
**2**	0.71	0.64	0.00	0.07	-
**3**	0.69	0.70	0.07	-	0.00
**4**	0.76	0.68	0.00	0.12	0.00
**5**	0.73	0.65	-	0.03	-
**6**	0.76	0.68	-	0.01	0.01
**7**	0.69	0.70	-	-	0.00

Table S4. Weights obtained from each data source when using PW with cuadratic windowing and KNN classifiers.
